# From 7-dehydrocholesterol to vitamin D_3_: Optimization of UV conversion procedures toward the valorization of fish waste matrices

**DOI:** 10.1016/j.fochx.2024.101373

**Published:** 2024-04-09

**Authors:** Yue Sun, Laura Alessandroni, Simone Angeloni, Erika Del Bianco, Gianni Sagratini

**Affiliations:** School of Pharmacy, Chemistry Interdisciplinary Project (ChIP), University of Camerino, 62032 Camerino, Italy

**Keywords:** Vitamin D_3_, Fish waste, UV conversion, By-products, HPLC-DAD

## Abstract

Vitamin D, a fat-soluble steroid, has increasingly taken a central role due to its crucial role in human health. It is estimated that about 40% of worldwide population are vitamin D deficient. The fish industry produces significant quantities of waste daily, with consequent high environmental impact. The aim of this work is to place a first brick for the fish waste reuse as a source of vitamin D_3_ extracts to be used for nutraceutical purposes. For this purpose, an UV conversion method for transforming the 7-dehydrocholesterol, highly present in fish, in vitamin D_3_ has been optimized. The UV wavelength, exposure time, temperature, stirring, and UV intensity were optimized using a surface response design tool. The optimized treatment was applied to five fish species with different fat percentages and the results were very promising reaching vitamin D_3_ levels >10 times higher than the pre-treatment ones.

## Highlights

• Fish can be considered good sources of 7-dehydrocholesterol, the vitamin D3 precursor.
• Optimization of 7-dehydrocholesterol to vitamin D3 UV conversion method.
• The most performing UV treatment was applied to five different fish species.
• The optimized method significantly increased the vitamin D3 content in the fishes.
• Reuse of fish waste as vitamin D3 source reducing environmental impact.

## 1 Introduction

Vitamin D is a fat-soluble vitamin that regulates calcium homeostasis and is vital for bone and neuromuscular system health (Sun, Nzekoue, Vittori, Sagratini, & Caprioli, 2022). Its main function is the development, growth, and maintenance of a healthy skeleton from birth until death. It accomplishes this by increasing the efficiency of the intestine to absorb dietary calcium (Holick, 2003). Other functions of vitamin D are related to various physiological processes that may influence the onset of numerous pathologies like cardiovascular and neurodegenerative diseases, rheumatological diseases, fertility, cancer, diabetes, and chronic fatigue condition (Di Molfetta, Bordoni, Gabbianelli, Sagratini, & Alessandroni, 2024).

Vitamin D deficiency is becoming increasingly prevalent around the world (Lips, de Jongh, & van Schoor, 2021). Recent observational data proved that ∼40% of Europeans are vitamin D deficient, and 13% are severely deficient (Amrein et al., 2020). According to previous studies, chronic vitamin D deficiency may have serious adverse consequences, including rickets in children, osteoporosis, increased risk of hypertension, multiple sclerosis, cancers of the colon, prostate, breast, and ovary, and type 1 diabetes (Barrea et al., 2022; Rihal, Khan, Kaur, & Singh, 2023).

Structurally, vitamin D naturally occurs in two forms, vitamin D_2_ (ergocalciferol) and vitamin D_3_ (cholecalciferol). The D_2_ form is specific of vegetable matrices while the D_3_ for in animal species (Cardwell, Bornman, James, & Black, 2018). In humans, it can be endogenously synthesized in the skin during sunlight exposure. More specifically, ultraviolet light (UV) can convert the 7-dehydrocholesterol (7-DHC) to pre-vitamin D_3_ in the upper layers of the skin, which is then converted into vitamin D_3_. A more-than-30-min sun exposure results in the opening of the 7-DHC B ring to generate pre-vitamin D_3_, which subsequently undergoes a thermal isomerization to D_3_ (cholecalciferol) (Slominski, Tuckey, Jetten, & Holick, 2023). During the 7-DHC conversion, overexposure was assessed to increase the production of pre-vitamin D_3_ in different metabolites such as lumisterol, tachysterol, suprasterols and 5,6 trans-vitamin D (Gatti, 2019). For many years, these secondary products were considered inactive isomers of vitamin D, but their biological activity and metabolism were investigated by recent studies, opening a new chapter in the field of endogenous sterols (Slominski et al., 2020; Slominski et al., 2022; Żmijewski, 2022). Vitamin D_3_ is hydroxylated in 25(OH)D_3_ (calcidiol) and then metabolized in the liver and kidney to the active form called 1α,25-dihydroxyvitamin D (calcitriol) which, binding the vitamin D receptor (VDR), can explicate its biological functions (Gombart, Pierre, & Maggini, 2020).

Naturally, only few foods contain Vitamin D, being mushrooms and reindeer lichen, for D_2_ form, and fatty fish (mackerel, salmon, sardines) and their liver oils, for D_3_ (Benedik, 2021). Earlier studies suggested that vitamin D_3_ showed a more effective absorption and bioavailability than vitamin D_2_ (Heaney, Recker, Grote, Horst, & Armas, 2011; Romagnoli et al., 2008). In fact, vitamin D_3_ was reported to be able to increase the total 25(OH)D blood concentration more than vitamin D_2_ (Di Molfetta et al., 2024; Lehmann et al., 2013).

To oppose vitamin D_3_ deficiency, comprehensive prevention strategies including pharmacological (supplementation) but also non-pharmacological treatment such as education, food fortification or lifestyle advice, have been proposed (Krasniqi, Boshnjaku, Ukëhaxhaj, Wagner, & Wessner, 2024). In this context, finding new sources of vitamin D or at least optimizing the use of already known ones, e.g., fish, could be valuable approaches. Nowadays, fishery, aquaculture and fish processing industry are involved in the production of daily significant quantities of waste with consequent high environmental impact (Coppola et al., 2021; Karkal & Kudre, 2020). This precious food is often discarded in percentages between 20 and 80% because it does not meet the market specifications (Lakra & Krishnani, 2022).

To the best of our knowledge, the production of vitamin D_3_ has been poorly studied in fish using UV radiation although 7-DHC occurs in fish tissues at a high level (Mgbechidinma et al., 2023; Pierens & Fraser, 2015). The scarce scientific literature mainly focuses on vitamin D_3_ production without planning an experimental optimization of all the parameters involved and without giving attention to the by-products that are certainly produced (Hidaka, Suzuki, Hayakawa, Okazaki, & Wada, 1989; Murthy, Phadke, Jeyakumari, & Vijayakumar, 2023; Sunitarao & Raghuramulu, 1997) or focuses on the irradiation of alive fish to obtain vitamin D_3_ rich fish meat (Ding, Jin, Fu, Zhang, & Guan, 2019). In this scenario, fish waste can be considered a great natural substrate available for the production of vitamin D_3_-rich extracts that can be further used in food functionalization or supplement formulation. Therefore, this study aimed to develop a reliable vitamin D_3_ production system based on not fish-species specific 7-DHC conversion toward future application on fish waste. Irradiation time, temperatures, lamp-sample distance and wavelength intensities, agitation and possible photoisomers production were investigated through a central composite design technique and further empirical tests. Thus, the optimized conditions were applied on different freeze-died fish matrices and the vitamin D_3_ formation was quantified together with the photoisomer and 7-DHC to evaluate the applicability of the newly developed approach on various fish species and, potentially, on fish waste. Therefore, the present study provides a starting point for the reuse of fish waste suggesting potential strategies for its sustainable utilization together with enhancing its nutritional value.

## 2 Materials and methods

### 2.1 Standards and reagents

Analytical standards of cholecalciferol (vitamin D_3_) (VD3; analytical standard; CAS number: 67–97-0), ergocalciferol (vitamin D_2_) (VD2; Pharmaceutical Secondary Standard; CAS number: 50–14-6), 7-dehydrocholesterol (7-DHC; ≥95.0% (HPLC); CAS number: 434–16-2), and Dihydrotachysterol (DHT; analytical standard, CAS number: 67–96-9) were supplied by Sigma Aldrich (Milan, Italy). Stock solutions were prepared at 1000 μg/mL by dissolving 10 mg of each standard in 10 mL of ethanol (EMSURE® Reag. Ph Eur; Merck, Darmstadt, Germany) and stored at −20 °C. Working solutions of different concentrations were daily prepared by appropriate dilution of the stock solutions with methanol. LC-MS-grade methanol was supplied by Merck (Darmstadt, Germany) while ultrapure water was obtained from a Milli-Q SP water system (Millipore, Bedford, MA). All the other solvents and reagents were analytical grade. Captiva PTFE 13 mm, 0.45 μm syringeless filter was bought from Agilent Technology (Santa Clara, CA, USA).

### 2.2 Experimental design for the optimization of conversion conditions

To optimize the irradiation conditions to increase the conversion yields of 7-DHC into vitamin D3, several tests were performed on 50 mL of 7-DHC standard ethanolic solution (10 mg/L), as reported by Nzekoue and colleagues, using a CN-6 irradiation chamber (Vilber Lourmat, France) (Nzekoue, Sun, Caprioli, Vittori, & Sagratini, 2022). Firstly, to assess the best wavelength, triplicate experiments were performed irradiating the 7-DHC solution at 254 nm (UVC) and 312 nm (UVB) at room temperature for 30 min. Then, a Design of Experiment tool of XLSTAT software (version 2023.1.4.1408) was used in Box-Behnken design mode to prepare the experimental plan of this work. Temperature and UV exposure time were set as variables. The experiments were performed according to the resulting experimental design (central composite design) combining temperature values from 20 °C to 70 °C and UV exposures from 30 to 90 min. After reaching the best combination of temperature and UV exposure time, further variables were monitored as the effect of agitation during the conversion and the UV intensity relatively to the lamp-sample distance (3 cm, 6 cm, 11 cm, 15 cm). The UV light intensity was measured and monitored using a UV light meter with a narrow band spectrum and high precision (1 μW/cm^2^). All the experiments were performed in triplicate and the concentration of analytes was monitored after each repetition.

### 2.3 HPLC-DAD quantification of vitamin D_3_, 7-DHC and DHT

The content of the VD3, the residual 7-DHC and the formation of a reaction by-product (photoisomer), i.e., DHT, were simultaneously quantified after each experiment using VD2 as an internal standard. After each treatment, the 10 mg/L ethanol from the standard solution of 7-DHC was evaporated using a N_2_ drying method; the analytes were resuspended in 1 mL of MeOH and filtered through a 0.45 μm PTFE filter. The analysis was then performed using a 1260 Infinity II high-performance liquid chromatography instrument coupled with a diode array detector (HPLC-DAD) (Agilent Technologies, Santa Clara, CA, USA). The separation of the analytes was obtained using a Gemini C18 analytical column (250 × 3.0 mm, 5 μm) set at 40 °C. The elution was performed at a flow rate of 1.0 mL/min in isocratic mode with 95% of methanol and 5% of water. The injection volume was 20 μL and the run time was 40 min. VD3 and 7-DHC were monitored at 265 nm and DHT at 254 nm (Fig. S1). VD3, 7-DHC, and DHT were confirmed and quantified using the analytical standards and calibration curves were obtained injecting different concentrations of analyte standard solutions. Calibration curves were plotted using the response factor against concentration. The response factor was calculated as the ratio between the area of the analyte and the area of the internal standard (VD2). Limit of Detection (LOD) and Limit of Quantification (LOQ) are reported in Table S1. These were experimentally estimated injecting low concentrations of the analyte standard solutions and measuring the signal-to-noise (S/N) ratio. A concentration giving a S/N ratio (height of peak/height of noise) of three was assigned to LOD while that of ten was LOQ.

### 2.4 Fish sample application

Different samples of fish were selected among the commercial species caught in Adriatic Sea and available, taking into account the different fat content: a fatty species being mackerel (*Scomber scombrus*), three medium fatty species being bluefish anchovy (*Engraulis encrasicolus*), sardine (*Sardina pilchardus*), and red mullet (*Mullus barbatus*) and a lean species being European hake (*Merluccius merluccius*). Whole fish samples were freeze-dried until weight stabilization using a LyovaporTM L-200 system (Buchi, Milan, Italy). For the extraction of 7-DHC, VD3 and DHT a previously published method was used with few modifications (Lu et al., 2007). Briefly, a total of 5 g of each freeze-dried fish sample was weighed, minced and 100 μL of 100 mg/L VD2 solution was added as internal standard. The alkaline digestion step was prepared adding 12 mL NaCl 1% solution, 4 mL ascorbic acid 1% solution, 18 mL of ethanol and 4 mL KOH 1:1 aqueous solution. The reaction was performed for 90 min at 65 °C in a water bath and then stopped using an ice bath for 30 min. The analytes were extracted with 3 mL of hexane, mixing, centrifuging and collecting the supernatant. The extraction was repeated three times to finally obtain 9 mL of hexane extract for each sample. Hexane was removed under nitrogen flow until drying and the extracts were reconstituted with 1 mL of MeOH to be filtered and injected in HPLC-DAD. The same procedure was applied to UV-treated samples. All the samples were performed in triplicate.

### 2.5 Statistical analysis

The Design of Experiment was prepared in Box-Behnken designs mode and analyzed through XLSTAT software (version 2023.1.4.1408). All experimental data were subject to one-way analysis of variance (ANOVA) and are reported as triplicate average values and standard deviations.

## 3 Results and discussion

### 3.1 Optimization of 7-DHC conversion in vitamin D3

The study's primary goal was to discover the most efficient UV wavelength to allow the conversion of 7-DHC in VD3; for this purpose, two wavelengths were tested, namely UVC at 254 nm and UVB at 312 nm, keeping the other parameters constant. A 30-min irradiation experiment on 7-DHC ethanol solution was performed at room temperature. Then, VD3, DHT as a by-product, and residual 7-DHC were quantified. The most efficient wavelength was 312 nm as it allowed to obtain 4.14 ± 0.08 μg/mL of VD3 against the 2.20 ± 0.24 μg/mL obtained after 254 nm irradiation. The residual 7-DHC was 480.3 ± 5.794 μg/mL and 477.4 ± 13.94 μg/mL for 312 nm and 254 nm respectively.

**Fig. 1:**
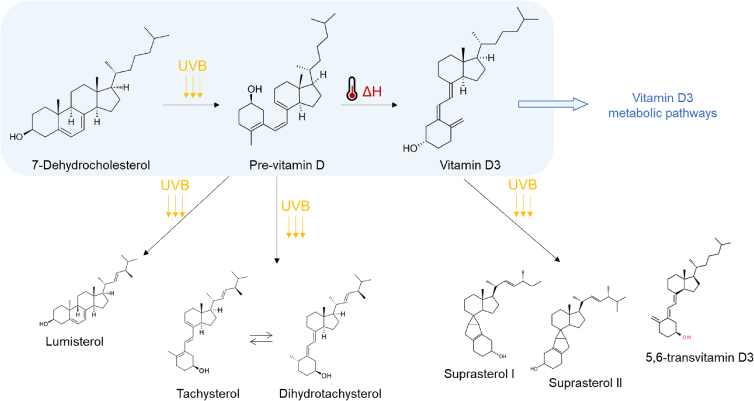
Vitamin D3 synthesis from 7-dehydrocholesterol and formation of by-products.

**Fig. 2:**
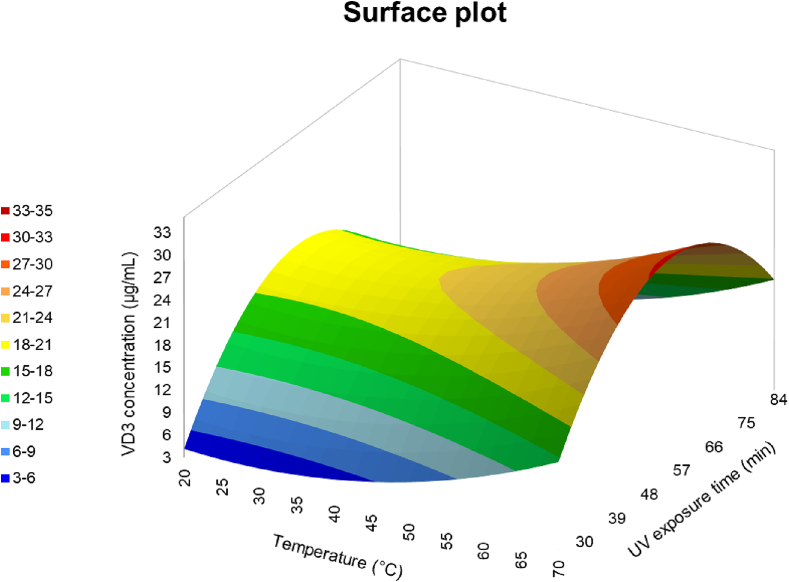
Surface plot resulted from the Design of Experiment to evaluate the best UV exposure time and temperature.

After that, to optimize the exposure time and the temperature, a Design of Experiment was prepared as explained in section 2.2. The results of the performed experiments are reported in a surface plot in Fig. 2. The plot shows the amount of generated VD3 at different temperatures during different times of irradiation. The level of VD3 increased proportionally with the temperature with a maximum value at 70 °C. The UV exposure time, instead, is not directly proportional to VD3 synthesis, in fact, the maximum yield was registered after 60 min of exposure, followed by a decrease in conversion for longer periods. Combining the results of time and temperature maximum points, it was possible to synthetize 32.72 ± 0.12 μg/mL of VD3. According to the VD3 conversion mechanism (Fig. 1), the lower yield obtained after longer UV exposure is due to the formation of by-products such as DHT. The slight reduction in vitamin D_3_ content related to >60 min of irradiation could be due to the irreversible production of secondary products by dimerization and ring cleavage (Jasinghe & Perera, 2006). A previous work reported that a prolonged irradiation time can lead to a quasi-equilibrium mixture of isomers with relative amounts depending on the spectrum and length of irradiation (Webb, 2006). Moreover, a prolonged irradiation also contributes to the creation of oxidative atmosphere resulting in VD3 photo-degradation (Vayalil, Elmets, & Katiyar, 2003).

**Fig. 3:**
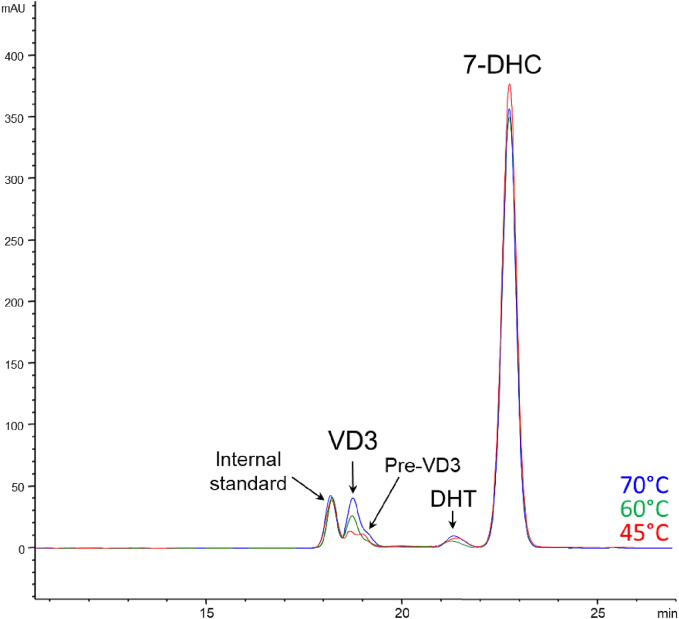
Effect of temperature. Overlap of resulting chromatograms from three 30-min irradiation experiments at different temperatures: 45 °C (red), 60 °C (green) and 70 °C (blue). (For interpretation of the references to colour in this figure legend, the reader is referred to the web version of this article.)

**Fig. 4:**
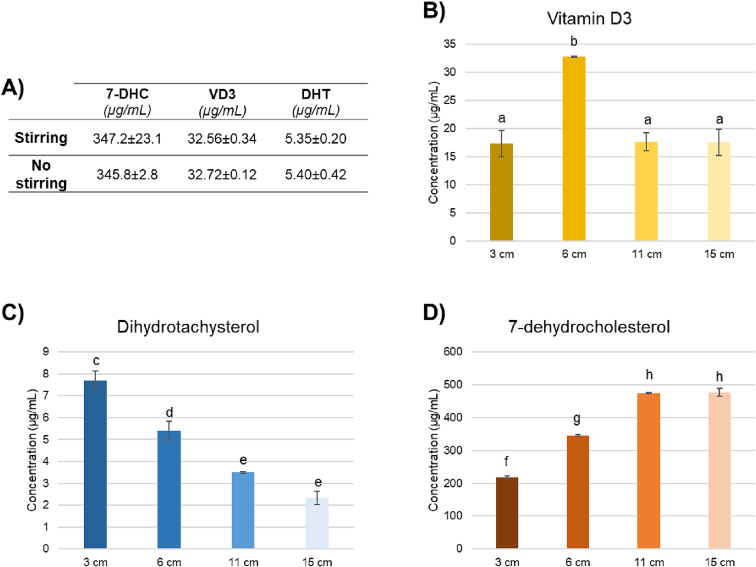
Effect of stirring and lamp-sample distance. A) Average concentration values of stirring and no-stirring experiments. B) Effect of lamp-sample distance on VD3, C)DHT and D) residual 7-DHC concentrations. Different letters in each graph report the statistically significant differences.

**Fig. 5:**
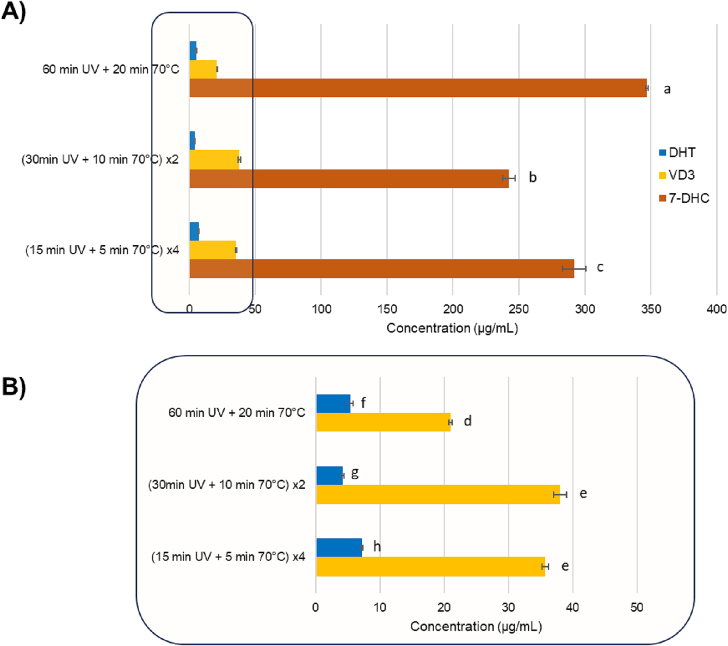
Splitting process effect. A) Overview of analytes in-vial concentrations. B) zoom-in to appreciate the VD3 and DHT formation. Different letters in each graph report the statistically significant differences in the same analytes among different splitting conditions.

**Fig. 6:**
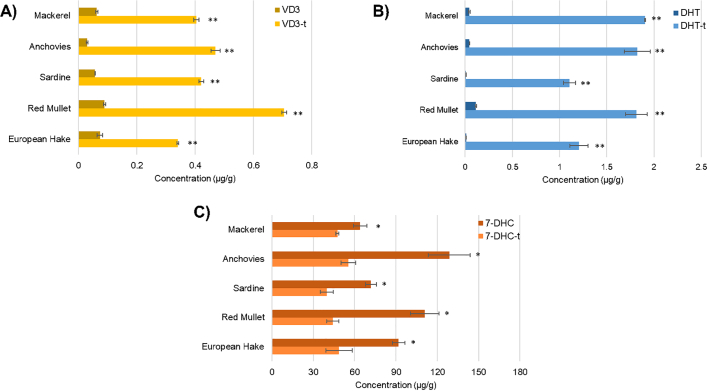
Effect of optimized method on real fish samples matrices. A) Concentration of VD3 before (VD3) and after the treatment (VD3-t) expressed in μg/g of freeze-dried fish. B) Concentration of DHT before (DHT) and after the treatment (DHT-t) expressed in μg/g of freeze-dried fish. C) Concentration of 7-DHC before (7-DHC) and after the treatment (7-DHC-t) expressed in μg/g of freeze-dried fish. In each graph the average values of triplicate are reported with standard deviations and the statistical difference between the values before and after the treatment are highlighted with asterisks (*:*p* < 0.05 and **p < 0.01). Values are reported in Table 1.

The concentration of DHT as a by-product was monitored during the experiments. Converting at 20, 45 and 60 °C, the concentrations of DHT were around or lower than 4 μg/mL. Working at 70 °C, a higher amount of DHT was produced in the 45 min procedure (6.56 ± 0.17 μg/mL) while slightly lower values were achieved in 60 and 90 min experiments (5.40 ± 0.42 μg/mL and 4.70 ± 0.62 μg/mL, respectively).

Moreover, in low-temperature experiments such as 45 °C the formation of an additional peak was noticed in the chromatogram (Fig. 3). As the peak was not present in the chromatograms of samples irradiated and treated at higher temperatures, the formation of previtamin D_3_ was hypothesized. In fact, according to Fig. 1, it is created by UV-B irradiation but still needs energy to thermoisomerize to VD3. Alternatively, this intermediate, can also further photoisomerize producing inert molecules as tachysterol and lumisterol, or return in 7-DHC form (Hurst, Homer, & Mellanby, 2020; Jasinghe & Perera, 2006). This statement will be further discussed in section 3.1.2. In physiological conversion, the heat isomerization of previtamin D_3_ to VD3 takes place in the skin and takes several hours (Webb, 2006). This statement further confirms our hypothesis that the second unknown peak could effectively be the previtamin D3, as it is reported to be a quite-stable intermediate, and the HPLC-DAD injections were always performed immediately after each experiment without any storage time.

#### 3.1.1 Effect of lamp-sample distance and stirring

This study aimed to further optimize the process evaluating the impact of stirring and the distance between the solution and the irradiation source. For this purpose, after establishing the optimal conversion parameters being a wavelength of 312 nm, irradiation time of 60 min and heating at 70 °C, two further experiments were performed. Firstly, the 7-DHC solution was stirred during the irradiation and the results were compared with the non-stirring procedure. Results are reported in Fig. 4A. Distance-related experiments were carried out in triplicates at various distances (3, 6, 11, and 15 cm) related to different UV intensity (0.48 mW/cm^2^, 0.31 mW/cm^2^, 0.16 mW/cm^2^, 0.1 mW/cm^2^, respectively) based on the capabilities of the device and results are reported in Fig. 4B, C, D. No statistically significant differences were found neither in VD3 and DHT formation nor in residual 7-DHC concentration, so no stirring would be used in further optimization tests. On the other hand, the sample's distance from the light source was discovered to have a significant influence on the conversion outcome. A significantly higher concentration of VD3 was found in samples irradiated at 6 cm (33.18 ± 0.81 μg/mL) (Fig. 4B). Examining the concentration variations of 7-DHC and DHT at different distances (Fig. 4C and D), the relationship between the conversion and UV intensity is visible. Indeed, in samples closer to the lamp the synthesis of DHT as an over-photoisomerization product is significantly higher, while the UV-B intensity that reaches the sample is not enough to allow a good conversion when the samples are placed at 11 and 15 cm. In conclusion, being too close to the source leads to a large production of over-irradiation by-products while too long distance does not provide enough energy to achieve a conversion as the strength of ultraviolet light drops as the distance from the UV lamp increases.

#### 3.1.2 Effect of process splitting strategies

At this level, the optimized method involves 60 min of UV-B irradiation and 70 °C of heating with a lamp-sample distance of 6 cm. Starting from this, we hypothesize that, according to the conversion and isomerization mechanism (Fig. 1), the yield could be higher by splitting the procedure in two: an initial irradiation phase that produces pre-vitamin D3, followed by a subsequent temperature rise to aid in the conversion into vitamin D3 (Holick & Slominski, 2024). Therefore, three hypotheses of a two-step protocol were structured and tested: (1) 7-DHC irradiation for 60 min, and then heated at 70 °C for 20 min. (2) 7-DHC irradiation for 30 min, and then heated at 70 °C for 10 min, repeating the procedure twice. (3) 7-DHC irradiation for 15 min and heating at 70 °C for 5 min, repeating the procedure four times. All the hypothesis were tested in triplicate.

The resulting concentrations are reported in Fig. 5. In conclusion, the splitting strategy resulted to be effective, in fact, splitting the total exposure and heating time in cycles resulted in higher VD3 (Fig. 5B). Although there is no statistically significant difference between the two splitting conditions (*p* *=* *0.08*), the amount of VD3 converted in the 2-cycles and 4-cycles experiments resulted statistically significant higher than the non-split ones (*p* *<* *0.01* in both cases). Moreover, an important difference was reported in by-product formation (*p* *<* *0.01*) between the 2-cycles and the 4-cycles strategy (4.21 ± 0.18 μg/mL and 7.21 ± 0.17 μg/mL of DHT, respectively). This could suggest that the photoproduced pre-VD3, heated just for 5 min, does not receive enough energy to thermoisomerize to VD3, therefore, when it is put back under UVB light it converts into by-product such as DHT. As mentioned before, the intermediate, if not properly heated, can further produce by-product or return in 7-DHC form (Jasinghe & Perera, 2006; Sunitarao & Raghuramulu, 1997). Accordingly, in 4-cycles strategy, not only the DHT concentration is higher than in 2-cycles, but also the residual 7-DHC resulted in higher and statistically significant values (292.04 ± 8.76 μg/mL and 242.3 ± 4.569 μg/mL respectively) (Fig. 5A). Concluding, the best conversion conditions were 30 min of 312 nm UV irradiation at 6 cm lamp-sample distance and 70 °C heating for 10 min, repeating the procedure twice.

### 3.2 Fish sample application results

The last aim of this research was to apply the optimized method to a real fish matrix. To achieve this, the procedure was applied to five different previously freeze-dried fish species. Firstly, VD3, 7-DHC and DHT were quantified in not-treated samples, then the optimized procedure of conversion was applied and the changes in analytes concentration were monitored. The analytes were extracted and quantified as reported in 2.4, 2.3, respectively. Even if the pre-treatment VD3 concentrations were very low, mullet and hake resulted to be richer (0.089 ± 0.004 μg/g and 0.073 ± 0.010 μg/g of freeze-dried fish, respectively) among the selected fish species. The lowest amount of VD3 was found in anchovies being lower the LOQ value. Bluefish are considered to be an excellent dietary source of VD3 because of their very oily nature. However, according to the obtained values and to Lu and colleagues conclusions, the concentration of this vitamin is not always this high. In fact, VD3 values were reported as more than three times higher in cod, as lean fish, than in blue fish (Lu et al., 2007). Therefore, fatty fish should not be addressed as the only source of vitamin D3 as the lean species emerged to be a better dietary source, together with a lower fat intake. Notably, other published data indicated that not only the fish species but also the living conditions can affect the vitamin D_3_ content in fish meat (Ding et al., 2019).

The concentrations of DHT were not quantifiable in sardines and hake, being lower the LOQ values, and very low in the other not-treated fish with 0.115 ± 0.007 μg/g of freeze-dried matrix as the highest value found in mullet samples. On the other hand, important values of VD3 precursor (7-DHC) concentration emerged.

After treatment, the amounts of VD3, DHT and residual 7-DHC were compared with the pre-treatment ones, as shown in Fig. 6 and Table 1. In all fish samples the final concentration of VD3 (VD3-t) was higher than in non-treated samples, with statistical significance (*p* *<* *0.01*). The same trend was reported for DHT as a reaction by-product. At the same time, the 7-DHC content decreased in all the fish samples with statistical significance (*p* *<* *0.05*).

**Table 1:** Results of quantifications of 7-DHC, VD3 and DHT before and after the treatment (‐t) of the tested fish matrices expressed in μg/g of freeze-dried fish. (n.q., not quantifiable, values lower the LOQ).

	7-DHC	7-DHC-t	VD3	VD3-t	DHT	DHT-t
Mackerel	64.05 ± 4.75	47.48 ± 1.05	0.063 ± 0.004	0.404 ± 0.009	0.044 ± 0.010	1.906 ± 0.004
Anchovies	128.67 ± 15.14	55.50 ± 5.28	n.q.	0.470 ± 0.015	0.045 ± 0.002	1.823 ± 0.137
Sardine	71.86 ± 4.72	39.76 ± 4.72	0.057 ± 0.001	0.421 ± 0.008	n.q.	1.104 ± 0.063
Red Mullet	111.09 ± 10.35	44.26 ± 4.43	0.089 ± 0.004	0.705 ± 0.008	0.115 ± 0.007	1.814 ± 0.114
European Hake	91.86 ± 9.59	48.66 ± 9.59	0.073 ± 0.010	0.341 ± 0.003	n.q.	1.207 ± 0.096

Initial evidences of 7-DHC conversion to VD3 in fish matrices were reported in 1997 (Sunitarao & Raghuramulu, 1997). In particular, the study focused on the exposing alive *Oreochromis mossambicus* (*Tilapia mossambica*) to UVB light (300 nm) for 15 h and subsequent quantification of VD3 in the meat of the sacrificed animal. The authors reported that UV exposure of fish samples resulted in 3-times higher VD3 levels and significantly lower 7-DHC than not-irradiated samples. However, in their study, no optimization of any conversion parameters was investigated.

A more recent study focused on UV exposure optimization parameters using alive juvenile salangidae, sardine and *hemiculter clupeoides* (Ding et al., 2019). The authors showed that the highest VD3 content (2.32 μg/g of dry fish) was found after a UVB irradiation related to the lowest detection of pre-VD3, tachysterol and other sterols. They tested the exposure time and lamp-sample distance resulting in non-statistically significant differences among 30, 60 and 90 min and 5 cm as the most effective. Even if their results are in line with the values that emerged in this paper, species-specific optimization on alive fish was carried out.

In conclusion, in fish samples lot of variables can interfere with the VD3 synthesis as they are complex matrices but the results of this work show that, although the high production of by-products, such as DHT, the on-standard optimized treatment, being not fish species-specific, has the potential to be implemented in an optic of reuse of multiple fish species waste as source of VD3 rich extracts.

## 4 Conclusions

The paper focuses on the optimization of a UVB irradiation process to convert the 7-DHC to VD3 in fish matrices by evaluating, for the first time, a high number of parameters. In addition, this study represents a first step to start thinking about taking advantage of the great amount of fish waste that is every day produced by the fishery industry as a source of rich extracts to be used in nutraceuticals, food supplementation and functionalization. These waste materials contain interesting nutrients, e.g., vitamin D3 and its precursors, which can be exploited for producing enriched extracts. Further studies are needed to refine the crucial point of extracts production. This could be investigated using green solvents or other extraction techniques, such as supercritical carbon dioxide. In this way, it would be possible to obtain safe extracts which are suitable for pharmaceutical and nutraceutical sectors.

## Funding information

The present work is part of the research activities developed within the project “VITADWASTE—Innovative and sustainable processes for the development of Vitamin D nutraceutical from fish waste: extraction, formulation and clinical study for the evaluation of its bioavailability and clinical equivalence” funded by MIUR—Ministero dell'Istruzione dell'Università e della Ricerca—PRIN: Progetti di Ricerca di Rilevante Interesse Nazionale, Bando 2022.

## CRediT authorship contribution statement

**Yue Sun:** Writing – original draft, Validation, Formal analysis, Investigation, Methodology. **Laura Alessandroni:** Writing – review & editing, Writing – original draft, Validation, Software, Investigation, Data curation, Conceptualization. **Simone Angeloni:** Formal analysis, Methodology, Validation, Writing – review & editing. **Erika Del Bianco:** Investigation, Conceptualization, Writing – original draft. **Gianni Sagratini:** Funding acquisition, Project administration, Resources, Supervision, Validation, Visualization, Writing – review & editing.

## Declaration of competing interest

The authors declare that they have no known competing financial interests or personal relationships that could have appeared to influence the work reported in this paper.

## Data Availability

Data will be made available on request.

## Appendix A Supplementary data

Figure S1. HPLC-DAD chromatograms of the analytical standards (10 mg/L), i.e., vitamin D3, 7-dehydrocholesterol, dihydrotachysterol and internal standard (VD2). VD3, VD2 and 7-DHC were monitored at 265 nm (in blue) and DHT at 254 nm (in green). Table S1. Limit of detection (LOD) and limit of quantification (LOQ) of the analytes. Image 1
