# Supplementary material for: From 7-dehydrocholesterol to vitamin D3: Optimization of UV conversion procedures toward the valorization of fish waste matrices
Source: Food Chem X. 2024 Apr 9;22:101373. doi: 10.1016/j.fochx.2024.101373 (PMC11021362; doi:10.1016/j.fochx.2024.101373)
Supplement: Supplementary file 1 — Figure S1. HPLC-DAD chromatograms of the analytical standards (10 mg/L), i.e., vitamin D3, 7-dehydrocholesterol, dihydrotachysterol and internal standard (VD2). VD3, VD2 and 7-DHC were monitored at 265 nm (in blue) and DHT at 254 nm (in green). Table S1. Limit of detection (LOD) and limit of quantification (LOQ) of the analytes. [file mmc1.docx]

**Figure S1.** HPLC-DAD chromatograms of the analytical standards (10 mg/L), i.e., vitamin D3, 7-dehydrocholesterol, dihydrotachysterol and internal standard (VD2). VD3, VD2 and 7-DHC were monitored at 265 nm (in blue) and DHT at 254 nm (in green).


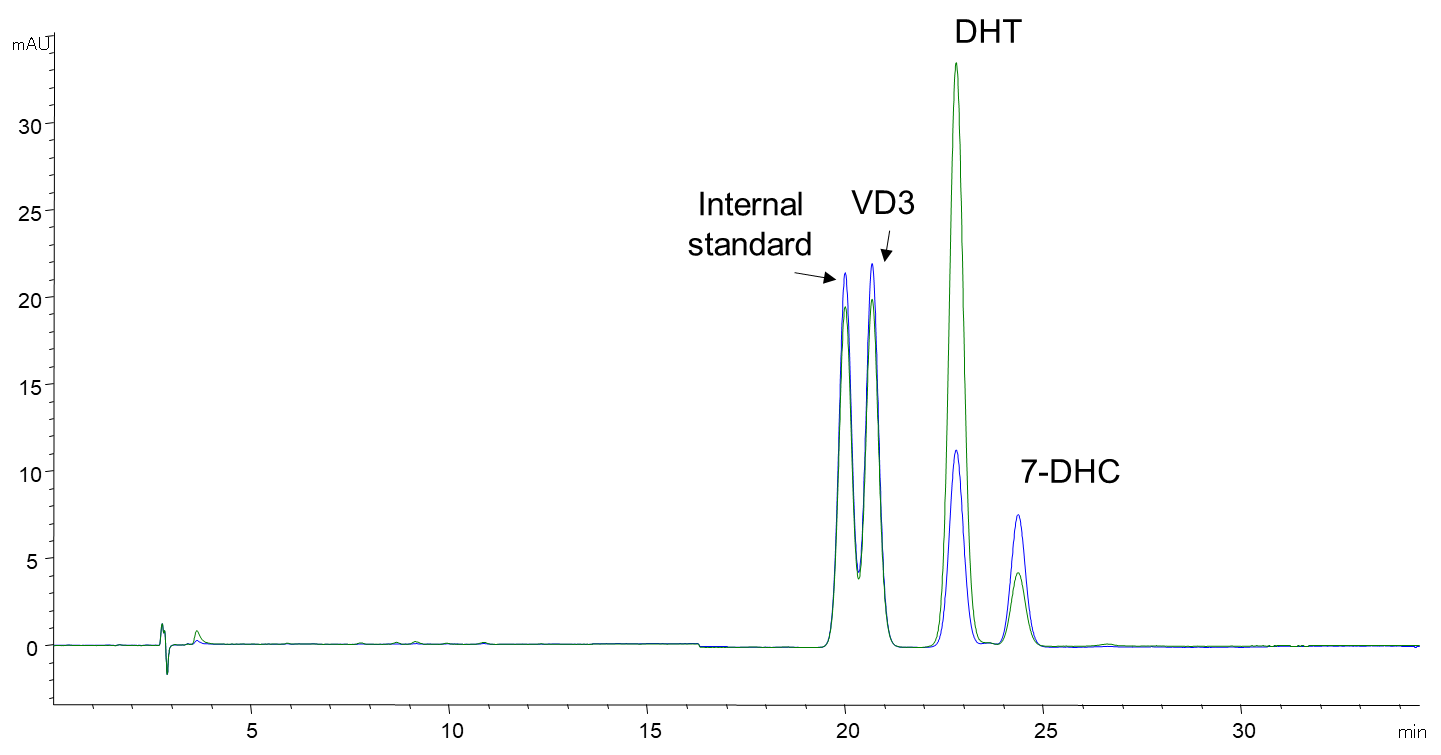


**Table S1.** Limit of detection (LOD) and limit of quantification (LOQ) of the analytes.

|  | LOD (mg/L) | LOQ (mg/L) |
| --- | --- | --- |
| 7-DHC | 0.165 | 0.550 |
| VD3 | 0.052 | 0.174 |
| DHT | 0.062 | 0.171 |
